# 610 From Flames to Facts: Unveiling Discrepancies in Burn Patient Documentation

**DOI:** 10.1093/jbcr/irae036.243

**Published:** 2024-04-17

**Authors:** Matthew L Boroditsky, Jenny Xiao, Fagun Jain, Sabina Dobrer, Erik N Vu, Anthony Papp

**Affiliations:** University of British Columbia, Vancouver, BC; WHRI/UBC, Vancouver, BC; VCHA, Vancouver, BC; Vancouver General Hospital, Vancouver, BC; University of British Columbia, Vancouver, BC; WHRI/UBC, Vancouver, BC; VCHA, Vancouver, BC; Vancouver General Hospital, Vancouver, BC; University of British Columbia, Vancouver, BC; WHRI/UBC, Vancouver, BC; VCHA, Vancouver, BC; Vancouver General Hospital, Vancouver, BC; University of British Columbia, Vancouver, BC; WHRI/UBC, Vancouver, BC; VCHA, Vancouver, BC; Vancouver General Hospital, Vancouver, BC; University of British Columbia, Vancouver, BC; WHRI/UBC, Vancouver, BC; VCHA, Vancouver, BC; Vancouver General Hospital, Vancouver, BC; University of British Columbia, Vancouver, BC; WHRI/UBC, Vancouver, BC; VCHA, Vancouver, BC; Vancouver General Hospital, Vancouver, BC

## Abstract

**Introduction:**

Comprehensive and accurate burn documentation is essential for initial and ongoing patient care. However, the challenge of inaccurate and incomplete records poses preventable risks. Our study evaluates burn documentation at a tertiary care burn centre, focusing on discrepancies between initial Emergency Department (ED) assessments and final evaluations by the Plastic Surgery Burn Consultant (PS). We hypothesize that the ED reports inaccurate and incomplete burn injury details, leading to differences in burn size and severity compared to PS assessments.

**Methods:**

We conducted a retrospective review of our provincial burn registry from January 1, 2016, to December 31, 2021. We included patients admitted for burns warranting a PS consultation, excluding isolated first-degree, ocular, and inhalational burns, and those not requiring burn unit admission. Data covering time, date, etiology, injury details, treatment, and follow-up were collected and compared between ED and PS records. Incomplete entries lacked burn-specific data points. Mann-Whitney and Kruskal-Wallis H tests were used to compare continuous outcomes, while Pearson’s Chi-Square test was employed for categorical outcomes. Wilcoxon’s Signed-Rank test was used to identify significant variations in TBSA estimates, with PS considered the "gold" standard. Statistical significance was set at p< 0.05.

**Results:**

358 patients were included, with most burns in male patients (76%) occurring at home and involving the head and neck. Burn etiology, circumstances, place, and anatomic location were well reported and consistent across PS and ED documentation. However, there were significant differences in TBSA estimates. The ED calculated a median TBSA of 20 (IQR: 19.8), while PS estimated a median TBSA of 14 (IQR: 16) (p< 0.0019). Notably, TBSA estimates for burns < 10% and 10-25% showed significant differences (p< 0.0001), tending toward overestimation by the ED. Deeper burns were consistently over-reported during initial ED assessments compared to the final PS determinations. Furthermore, 81% of the initial records were incomplete: 66% lacked initial treatment data, 49% missed TBSA, and 39% omitted burn depth.

**Conclusions:**

Significant discrepancies were appreciated in the initial ED documentation of burn injuries at our tertiary care burn centre, with overestimations in TBSA and burn depth. Over 80% of initial documentation was incomplete, with TBSA omitted in 49% of charts in both local and peripheral transfer consultations. There is a collective urgent need for enhanced awareness and education on the importance of accurate and comprehensive burn patient documentation.

**Applicability of Research to Practice:**

This research emphasizes the need for improved documentation strategies for burn patients seen in the acute care setting, raising the potential to venture into modalities such as artificial intelligence and electronic health record systems to enhance burn documentation.

